# Systematic optimization of gene expression of pentose phosphate pathway enhances ethanol production from a glucose/xylose mixed medium in a recombinant *Saccharomyces cerevisiae*

**DOI:** 10.1186/s13568-018-0670-8

**Published:** 2018-08-27

**Authors:** Yosuke Kobayashi, Takehiko Sahara, Satoru Ohgiya, Yoichi Kamagata, Kazuhiro E. Fujimori

**Affiliations:** 1 Bioproduction Research Institute (BPRI), National Institute of Advanced Industrial Science and Technology (AIST), 1-1-1 Higashi, Tsukuba, Ibaraki 305-8566 Japan; 2 Bioproduction Research Institute (BPRI), National Institute of Advanced Industrial Science and Technology (AIST), 2-17-2-1 Tsukisamu-higashi, Toyohira, Sapporo, Hokkaido 062-8517 Japan

**Keywords:** Bio-ethanol, Glucose/xylose co-fermentation, Xylose isomerase, Thermostability, *Saccharomyces cerevisiae*, *Kluyveromyces marxianus*

## Abstract

The pentose phosphate pathway (PPP) plays an important role in the synthesis of ribonucleotides and aromatic amino acids. During bioethanol production from cellulosic biomass composed mainly of d-glucose and d-xylose, the PPP is also involved in xylose metabolism by engineered *Saccharomyces cerevisiae*. Although the activities and thermostabilities of the four PPP enzymes (transaldolase: *TAL1*, transketolase: *TKL1*, ribose-5-phosphate ketol-isomerase: *RKI1* and d-ribulose-5-phosphate 3-epimerase: *RPE1*) can affect the efficiency of cellulosic ethanol production at high temperatures, little is known about the suitable expression levels of these PPP genes. Here, we overexpressed PPP genes from *S. cerevisiae* and the thermotolerant yeast *Kluyveromyces marxianus* either singly or in combination in recombinant yeast strains harboring a mutant of xylose isomerase (XI) and evaluated xylose consumption and ethanol production of these yeast transformants in glucose/xylose mixed media at 36 °C. Among the PPP genes examined, we found that: (1) strains that overexpressed *S. cerevisiae TKL1* exhibited the highest rate of xylose consumption relative to strains that overexpressed other PPP genes alone; (2) overexpression of *RKI1* and *TAL1* derived from *K. marxianus* with *S. cerevisiae TKL1* increased the xylose consumption rate by 1.87-fold at 24 h relative to the control strain (from 0.55 to 1.03 g/L/h); (3) the strains with XI showed higher ethanol yield than strains with xylose reductase and xylitol dehydrogenase and (4) *PHO13* disruption did not improve xylose assimilation under the experimental conditions. Together these results indicated that optimization of PPP activity improves xylose metabolism in genetically engineered yeast strains, which could be useful for commercial production of ethanol from cellulosic material.

**Electronic supplementary material:**

The online version of this article (10.1186/s13568-018-0670-8) contains supplementary material, which is available to authorized users.

## Introduction

Second-generation technology has been developed for bioethanol production from non-edible biomass composed of cellulose and hemicellulose, which are sugar polymers of d-glucose, d-xylose and l-arabinose (Naik et al. 2010) that can be hydrolyzed by cellulase and hemicellulase (Bhattacharya et al. 2015). The yeast *Saccharomyces cerevisiae* readily produces ethanol from d-glucose, but not from pentose sources such as d-xylose and l-arabinose (Young et al. 2010). In 1990, introduction of two *Scheffersomyces stipites* (formerly known as *Pichia stipites*) genes, *XYL1* and *XYL2,* encoding NADPH-dependent xylose reductase (XR) and NAD^+^-dependent xylitol dehydrogenase (XDH), respectively, into *S. cerevisiae* was shown to allow successful xylose fermentation (Kötter et al. 1990). Moreover, various genetic engineering trials with endogenous or exogenous genes have been applied to yeast transformants carrying xylose-metabolizing enzyme genes to improve ethanol production in xylose-containing media. For instance, xylose metabolism was markedly improved by deletion of the putative alkaline phosphate gene *PHO13* in *XLY1/XYL2* transformants (Van Vleet et al. 2008; Kim et al. 2015; Xu et al. 2016; Kobayashi et al. 2017). Overexpression of *HXT7,* a known xylose-permeable hexose transporter in *S. cerevisiae,* also improved xylose consumption (Gonçalves et al. 2014). However, critical issues remained for efficient xylose fermentation by *XYL1/XYL2* transformants such as accumulation of xylitol, sedoheptulose-7-phosphate and pyruvate, and depletion of fructose-1,6-bisphosphate because of insufficient capacity of the glycolytic pathway (Kötter and Ciriacy 1993). An imbalance of cofactors can also slow xylose metabolism (Van Vleet and Jeffries 2009). Previous studies reported that overexpression of non-oxidative pentose phosphate pathway (PPP) genes did not improve the xylose fermentation rate (Walfridsson et al. 1995; Johansson and Hahn-Hägerdal 2002; Matsushika et al. 2009).

Another strategy to enhance xylose metabolism in *S. cerevisiae* depended on the xylose isomerase (XI) gene derived from *Thermus thermophiles* (Walfridsson et al. 1996). The *S. cerevisiae* XI transformants had a better ethanol yield than did *XYL1/XYL2* since production inhibition by xylitol and cofactor imbalances was avoided (Brat et al. 2009). Meanwhile, deletion of either *PHO13* (Lee et al. 2014; Bamba et al. 2016) or the aldo–keto reductase gene *GRE3* (Träff et al. 2001), as well as introduction of an *HXT7* mutant (Reider Apel et al. 2016) improved xylose metabolism in the XI transformants.

In addition to introduction of the xylose metabolism gene, balanced expression of PPP genes is important for bioethanol production (Kuyper et al. 2005). Many studies revealed that overexpression of four non-oxidative PPP genes, transaldolase (*TAL1*), transketolase (*TKL1*), ribose-5-phosphate ketol-isomerase *(RKI1*) and d-ribulose-5-phosphate 3-epimerase (*RPE1*) increased ethanol production in medium in which d-xylose was the sole carbon source (Kuyper et al. 2005; Karhumaa et al. 2007; Lee et al. 2014; Qi et al. 2015). However, the effect of exogeneous expression of PPP genes on the rates of xylose consumption and ethanol production, particularly in a glucose/xylose-mixed medium, remained unclear, and no studies had addressed suitable activities for PPP enzymes that would enhance xylose metabolism. For ethanol production from multiple sugar sources, the metabolic flow of each sugar through the glycolytic pathway (GP) and PPP may be intricately involved in xylose metabolism. Furthermore, fermentation at high temperatures is preferable in the simultaneous saccharification and co-fermentation (SSCF) process because the optimal temperature for the cellulase reaction is much higher than that for fermentation by most ethanologenic microorganisms (Paulova et al. 2014; Kawaguchi et al. 2016).

To explore the optimal expression levels of PPP genes needed to maximize ethanol production from cellulosic sources, here we systematically investigated the effect of overexpression of single and multiple PPP genes derived from *S. cerevisiae* and the thermotolerant yeast *Kluyveromyces marxianus* in a *S. cerevisiae* strain harboring the XI gene that allows glucose/xylose co-fermentation at high temperatures. We explored differences in the favorable combination of PPP genes in *S. cerevisiae* between our previous study using XR-XDH transformants in medium having d-xylose as the sole sugar (Kobayashi et al. 2017) and this study using the XI transformants in the glucose/xylose-mixed medium.

## Materials and methods

### Media, general cultivation conditions, plasmid vectors, oligonucleotides and establishment of engineered *S. cerevisiae* strains

Yeast were cultivated in YPD medium (20 g/L Bacto™ peptone (BD Biosciences, Franklin Lakes, NJ, USA), 10 g/L Bacto™ yeast extract (BD Biosciences) and 20 g/L d-glucose (Sigma-Aldrich, St. Louis, MO, USA)) on YPD agar plates (20 g/L Bacto™ Agar, BD Biosciences) with Geneticin^®^ (Thermo Fisher Scientific, Waltham, MA, USA), Zeocin™ (Thermo Fisher Scientific) or Aureobasidin A (Takara Bio, Shiga, Japan) if necessary.

All yeast strains used in this study are listed in Table 1 with their genotypes. *S. cerevisiae* IR-2, a flocculating yeast isolated from waste water from a food-processing company in Indonesia, was used as the host strain (Kuriyama et al. 1985). A draft genome sequence of IR-2 was also available (Sahara et al. 2014, Accession Nos. BAUI01000001–BAUI01000322). Based up on the genome sequence, strain SS29 was first constructed as a reference strain. SS29 carries a deletion mutation of the *HO* gene, which maintains the haploid state, and in the *GRE3* gene, an intrinsic xylose reductase gene in *S. cerevisiae* that suppresses xylitol production (Träff et al. 2001). A mutant xylose isomerase gene derived from *Lachnoclostridium phytofermentans* Lp*XI*(T63I) (Brat et al. 2009, Accession No. LC390328) driven by the HSP12 promoter of *S. cerevisiae* was constructed in an AUR101 plasmid vector (Takara-Bio) with an additional copy of the *XKS1* gene from *Saccharomyces cerevisiae* IR-2 strain that provided compensatory xylulokinase activity. This plasmid was linearized by *Stu*I digestion and introduced at the *AUR1* locus of SS29 by single crossover homologous recombination. Selection with Aureobasidin A established the basal strain SS82. For chromosomal integration of exogeneous PPP gene(s), recipient vectors were constructed with the neomycin-resistant gene cassette *kan*MX6 and several combinations of PPP genes from *S. cerevisiae* and/or *K. marxianus* DMB1, which are codon-optimized for suitable expression in *S. cerevisiae,* and inserted between the TDH3 promoter and CYC1 terminator of *S. cerevisiae* (Kobayashi et al. 2017, Accession Nos. LC390324 for Km*RKI1*opt, LC390325 for Km*RPE1*opt, LC390326 for Km*TAL1* and LC390327 for Sc*TKL1*). The DNA fragments of the PPP gene cassettes including *kan*MX6 were amplified with primers to yield 50 bp overhangs for deletion of *GPD2* or *PHO13* loci via transformation (Additional file 1: Table S1). Yeast transformation was performed using a traditional LiOAc method (Gietz and Schiestl 2007). All DNA sequences of the constructed plasmid vectors and DNA fragments were verified by Sanger sequencing (Eurofins Japan, Tokyo Japan).

**Table 1:** *S. cerevisiae* strains used in this study

Code	Genotype, full description	
IR-2	*MATa*/α	(Kuriyama et al. 1985)
IR-2 2a-3-34A	*MAT*α *ho*Δ*::bleMX6*	Lab. stock
SS29	IR-2 2a-3-34A *gre3*Δ*::hphMX6*	(Seike et al. unpublished observations)
SS82	SS29 *AUR1*-*C::HSP12p*-*XI*_*mut*_-*CYC1t*-*XKS1*-*PGK1p*	(Seike et al. unpublished observations)
SS118	SS82 *gpd2*Δ*::kanMX6*	(Seike et al. unpublished observations)
YK184	SS82 *gpd2*Δ*::TDH3p*-*KmTAL1opt*-*CYC1t*-*kanMX6*	This study
YK185	SS82 *gpd2*Δ*::TDH3p*-*ScTKL1*-*CYC1t*-*kanMX6*	This study
YK183	SS82 *gpd2*Δ*::TDH3p*-*KmRKI1opt*-*CYC1t*-*kanMX6*	This study
YK186	SS82 *gpd2*Δ*::TDH3p*-*KmRPE1opt*-*CYC1t*-*kanMX6*	This study
YK223	SS82 *gpd2*Δ*::TDH3p*-*KmTAL1opt*-*CYC1t*-*TDH3p*-*ScTKL1*-*CYC1t*-*kanMX6*	This study
YK193	SS82 *gpd2*Δ*::TDH3p*-*KmRKI1opt*-*CYC1t*-*TDH3p*-*ScTKL1*-*CYC1t*-*kanMX6*	This study
YK224	SS82 *gpd2*Δ*::TDH3p*-*KmRPE1opt*-*CYC1t*-*TDH3p*-*ScTKL1*-*CYC1t*-*kanMX6*	This study
YK246	SS82 *gpd2*Δ*::TDH3p*-*KmRKI1opt*-*CYC1t*-*TDH3p*-*KmTAL1opt*-*CYC1t*- *TDH3p*-*ScTKL1*-*CYC1t*-*kanMX6*	This study
YK247	SS82 *gpd2*Δ*::TDH3p*-*KmRPE1opt*-*CYC1t*-*TDH3p*-*KmTAL1opt*-*CYC1t*- *TDH3p*-*ScTKL1*-*CYC1t*-*kanMX6*	This study
YK248	SS82 *gpd2*Δ*::TDH3p*-*KmRKI1opt*-*CYC1t*-*TDH3p*-*KmRPE1opt*-*CYC1t*- *TDH3p*-*KmTAL1opt*-*CYC1t* -*kanMX6*	This study
YK249	SS82 *gpd2*Δ*::TDH3p*-*KmRKI1opt*-*CYC1t*-*TDH3p*-*KmRPE1opt*-*CYC1t*- *TDH3p*-*ScTKL1*-*CYC1t*-*kanMX6*	This study
YK197	SS82 *gpd2*Δ*::TDH3p*-*KmRKI1opt*-*CYC1t*-*TDH3p*-*KmRPE1opt*-*CYC1t*- *TDH3p*-*KmTAL1opt*-*CYC1t*-*TDH3p*-*ScTKL1*-*CYC1t*-*kanMX6*	This study
YK001	IR-2 2a-3-12A *AUR1*-*C::PGK1p*-*SsXR*-*PGK1t*-*PGK1p*-*SsXDH*-*PGK1t*- *PGK1p*-*ScXKS1*-*PGKt*	(Kobayashi et al. 2017)
YK002	YK001 *pho13*Δ*::kanMX6*	(Kobayashi et al. 2017)
YK115	YK001 *pho13*Δ*::TDH3p*-*ScRKI1*-*CYC1t*-*TDH3p*-*ScRPE1*-*CYC1t*- *TDH3p*-*ScTAL1*-*CYC1t*-*TDH3p*-*ScTKL1*-*CYC1t*-*kanMX6*	(Kobayashi et al. 2017)
YK149	SS82 *pho13*Δ*::blaMX6*	This study
YK150	SS82 *pho13*Δ*::TDH3p*-*ScRKI1*-*CYC1t*-*TDH3p*-*ScRPE1*-*CYC1t*- *TDH3p*-*ScTAL1*-*CYC1t*-*TDH3p*-*ScTKL1*-*CYC1t*-*blaMX6*	This study

### Fermentation examination and quantitative analyses of fermentation performance by HPLC

Recombinant yeast strains were cultivated at 30 °C in YPD media under aerobic conditions by shaking at 150 rpm for 2 days until the stationary phase was reached. The cells were recovered and washed immediately with YPDX medium (20 g/L Bacto™ peptone, 10 g/L Bacto™ yeast extract, 85 g/L d-glucose and 35 g/L d-xylose) and diluted to an OD_600_ of 3 in 70 mL of YPDX medium. A silicon plug with needles and a three-way stopcock for sampling and aeration was used as a cap to aliquot the culture medium in 100 mL Erlenmeyer flasks. Fermentation studies were performed at 36 °C for up to 72 h with periodic sampling under micro-aerobic conditions.

The supernatants were collected by centrifugation, and the concentrations (g/L) of residual sugars, produced polyols, organic acids and ethanol in the fermentation media were measured using an HPLC equipped with a refractive index (RI) detector (JASCO Co., Tokyo, Japan). For separation of these chemical compounds, an Aminex HPX-87 H column (Bio-Rad, CA, USA) with a guard column (Cation H Cartridges 30 × 4.6 mm, Bio-Rad) was used under the following conditions: column oven temperature of 65 °C, 5 mM H_2_SO_4_ as the mobile phase buffer, and flow rate of 0.6 mL/min. Further details are described in our previous publication (Kobayashi et al. 2017).

## Results

### Effects of overexpressing a single PPP gene on glucose/xylose co-fermentation

**Fig. 1:**
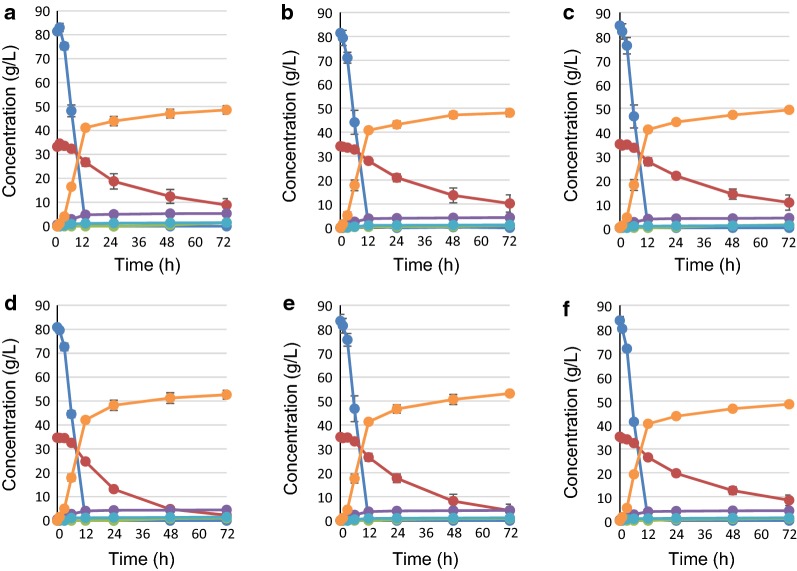
Fermentation profiles of strains expressing XI and overexpressing single PPP genes. Cells were initially inoculated at OD_600_ = 3 and cultivated in medium containing 85 g/L glucose and 35 g/L xylose for 72 h at 36 °C. **a** SS82, **b** SS118 (*GPD2*Δ), **c** YK184 (Km*TAL1opt*), **d** YK185 (Sc*TKL1*), **e** YK183 (Km*RKI1opt*), **f** YK186 (Km*RPE1opt*). Blue lines: glucose, Red lines: xylose, Green lines: xylitol, Purple lines: glycerol, Light blue lines: acetate, Orange lines: ethanol. Each value is an average of triplicate cultures

To determine the effect of overexpression of each PPP gene on glucose/xylose co-fermentation at 36 °C, batch-fermentation was first carried out under microaerobic conditions using strains YK184 (Km*TAL1opt*), YK185 (Sc*TKL1*), YK183 (Km*RKI1opt*) and YK186 (Km*RPE1opt*) and the fermentation profiles and metabolic characteristics were determined (Fig. 1, Table 2, Additional file 2: Table S2). Under microaerobic conditions, the control strain SS82 consumed approximately 85 g/L of d-glucose within 12 h, by which point the d-xylose concentration had slightly decreased. Across 72 h, the ethanol concentration gradually increased to reach 48.6 g/L. The xylose consumption rate over 24 h was about 0.60 g/L/h, and the ethanol yield over 72 h was 0.41 g-ethanol/g-total sugars (Table 2). Under this co-fermentation condition, consumption of d-xylose was incomplete at 72 h; an alternative calculation for the ethanol yield for 72 h was 0.44 g-ethanol/g-consumed sugars (calculation from Additional file 2: Table S2). Notably, 1.5 g/L xylitol was detected at 72 h even though *GRE3* was disrupted in SS82 (Fig. 1a, Additional file 2: Table S2). SS118, a control strain for overexpression of PPP gene(s), had a metabolic profile for co-fermentation that was similar to that of SS82 (Fig. 1b). The xylose consumption rate of YK185 expressing *S. cerevisiae TKL1* was 1.66-fold higher than that of SS118 at 24 h, whereas YK183 expressing *K. marxianus RKI1* was about 1.42-fold higher. Meanwhile, YK184 and YK186 showed no obvious improvement in xylose consumption or ethanol production rates (Figs. 1c–f). The amount of residual xylose for YK185 and YK183 was less than 5 g/L at 72 h. Therefore, the ethanol yields for these strains were clearly increased at 72 h (0.45 g-ethanol/g-total sugars) relative to the control strain SS118, while no change in the ethanol yields for YK184 and YK186 was observed (0.40–0.41 g-ethanol/g-total sugars). Together these results indicate that expression of *TKL1* and *RKI1* could contribute to increased xylose metabolism and concurrent increased ethanol yield.

**Table 2:** Comparison of fermentation performance

Strain	Y_eth_ (g/g)	C_xyl_ (g/L/h)		
	72 h	24 h	48 h	72 h
SS82	0.41 ± 0.01	0.60 ± 0.08	0.43 ± 0.04	0.34 ± 0.02
SS118	0.40 ± 0.02	0.55 ± 0.07	0.43 ± 0.06	0.33 ± 0.05
YK184	0.41 ± 0.01	0.55 ± 0.03	0.44 ± 0.04	0.34 ± 0.04
YK185	0.45 ± 0.03	0.90 ± 0.04	0.62 ± 0.02	0.45 ± 0.01
YK183	0.45 ± 0.02	0.72 ± 0.06	0.56 ± 0.06	0.43 ± 0.04
YK186	0.41 ± 0.01	0.64 ± 0.05	0.47 ± 0.04	0.37 ± 0.04
YK223	0.44 ± 0.01	0.73 ± 0.06	0.58 ± 0.04	0.44 ± 0.02
YK193	0.46 ± 0.01	0.94 ± 0.03	0.66 ± 0.02	0.46 ± 0.01
YK224	0.44 ± 0.00	0.71 ± 0.04	0.55 ± 0.03	0.42 ± 0.02
YK246	0.45 ± 0.01	1.03 ± 0.03	0.68 ± 0.01	0.48 ± 0.01
YK247	0.43 ± 0.01	0.76 ± 0.08	0.58 ± 0.04	0.44 ± 0.01
YK248	0.43 ± 0.00	0.82 ± 0.01	0.61 ± 0.00	0.44 ± 0.00
YK249	0.44 ± 0.02	0.83 ± 0.02	0.61 ± 0.02	0.45 ± 0.01
YK197	0.42 ± 0.01	0.41 ± 0.08	0.33 ± 0.06	0.26 ± 0.04
YK001	0.39 ± 0.01	0.68 ± 0.08	0.44 ± 0.07	0.31 ± 0.05
YK002	0.38 ± 0.00	0.62 ± 0.03	0.40 ± 0.07	0.28 ± 0.06
YK115	0.35 ± 0.01	0.32 ± 0.13	0.17 ± 0.07	0.13 ± 0.04
YK149	0.40 ± 0.00	0.61 ± 0.02	0.43 ± 0.01	0.33 ± 0.00
YK150	0.40 ± 0.01	0.50 ± 0.02	0.36 ± 0.03	0.29 ± 0.03

### Effects of overexpressing multiple PPP genes on glucose/xylose co-fermentation

**Fig. 2:**
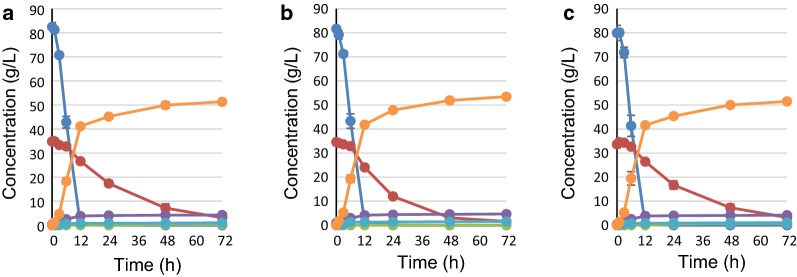
Fermentation profiles of strains expressing XI and overexpressing two PPP genes. Cells were initially inoculated at OD_600_ = 3 and cultivated in medium containing 85 g/L glucose and 35 g/L xylose for 72 h at 36 °C. **a** YK222 (*KmTAL1opt*-*ScTKL1*), **b** YK193 (Km*RKI1opt*-Sc*TKL1*), **c** YK223 (Km*RPE1opt*-Sc*TKL1*). Blue line: glucose, Red line: xylose, Green line: xylitol, Purple line: glycerol, Light blue line: acetate, Orange line: ethanol. Each data point is an average of triplicate cultures

**Fig. 3:**
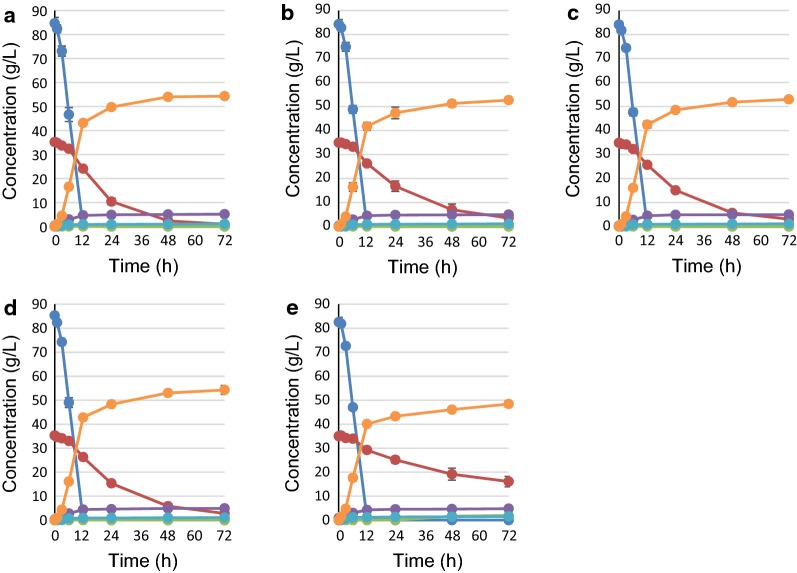
Fermentation profiles of strains expressing XI and combinatorial expression of multiple PPP genes. Cells were initially inoculated at OD_600_ = 3 and cultivated in a medium containing 85 g/L glucose and 35 g/L xylose for 72 h at 36 °C. **a** YK246 (Km*RKI1opt*- Km*TAL1opt* -Sc*TKL1*), **b** YK247 (Km*RPE1opt*-*KmTAL1opt*-*ScTKL1*), **c** YK248 (Km*RKI1opt*-Km*RPE1opt*-*KmTAL1opt*), **d** YK249 (Km*RKI1opt*-Km*RPE1opt*-Sc*TKL1*), **e** YK197 (Km*RKI1opt*-Km*RPE1opt*-Km*TAL1opt*-Sc*TKL1*). Blue line: glucose, Red line: xylose, Green line: xylitol, Purple line: glycerol, Light blue line: acetate, Orange line: ethanol. Each data point is an average of triplicate cultures

**Fig. 4:**
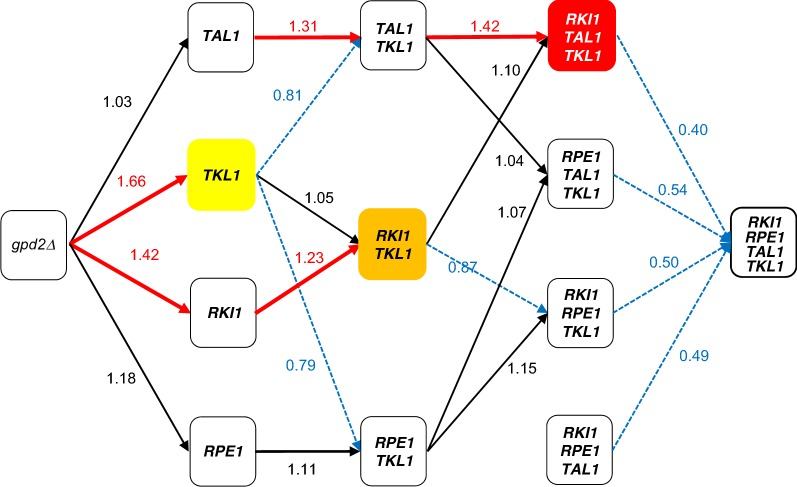
Successive changes in d-xylose consumption by introduction of each PPP gene. Black line: increased by less than 1.2-fold, Blue and dashed line decreasing, Red thick line: increased by more than 1.2-fold. Colored panels: gene combination with highest consumption rate in single (Yellow), double (Orange) and triple (Red) of PPP gene(s), respectively

Based on the results described above, the fermentation profiles and metabolic characteristics of strains YK223 (*gpd2*Δ::Km*TAL1opt*-Sc*TKL1*), YK193 (*gpd2*Δ::Km*RKI1opt*-Sc*TKL1*) and YK224 (*gpd2*Δ::Km*RPE1opt*-Sc*TKL1*) that co-expressed two PPP genes; and YK246 (*gpd2*Δ::Km*RKI1opt*- Km*TAL1opt*-Sc*TKL1*), YK247 (*gpd2*Δ::Km*RPE1opt*-Km*TAL1opt*-Sc*TKL1*), YK248 (*gpd2*Δ::Km*RKI1opt*-Km*RPE1opt*-Km*TAL1opt*) and YK249 (*gpd2*Δ::Km*RKI1opt*-Km*RPE1opt*-Sc*TKL1*) that co-expressed three PPP genes; as well as YK197 that co-expressed all PPP genes (*gpd2*Δ::Km*RKI1opt*-Km*RPE1opt*-Km*TAL1opt*-Sc*TKL1*) were examined on glucose/xylose co-fermentation at 36 °C (Figs. 2, 3, Table 2, Additional file 2: Table S2). The xylose consumption rates at 24 h for strains YK223 (co-express Km*TAL1* and Sc*TKL1*), YK193 (co-express Km*RKI1* and Sc*TKL1*) and YK224 (co-express Km*RPE1* and Sc*TKL1*) increased by about 1.32-, 1.71- and 1.29-fold, respectively, relative to SS118. The addition of Sc*TKL1* to singly expressed Km*TAL1opt* (YK184) and Km*RKI1opt* (YK183) increased xylose consumption rates at 24 h by about 1.31- and 1.23-fold, respectively, whereas no increase was seen for co-expression of Sc*TKL1* with Km*RKI1opt* (YK193). The xylose consumption rates at 24 h for strains co-expressing three PPP genes (YK246 (Km*RKI1*- Km*TAL1*- Sc*TKL1*), YK247 (Km*RPE1*- Km*TAL1*- Sc*TKL1*), YK248 (Km*RKI1*- Km*RPE1*- Km*TAL1*) and YK249 (Km*RKI1*- Km*RPE1*- Sc*TKL1*) increased by 1.87-, 1.38-, 1.49- and 1.51-fold, respectively, compared to SS118. The xylose consumption rate at 24 h for YK246 increased by 1.42- and 1.10-fold relative to that for YK223 and YK193, respectively. Meanwhile, the xylose consumption rate at 24 h for YK247 did not increase compared to that for YK223 or YK224. The xylose consumption rate for YK249 at 24 h increased by 1.17-fold over that for YK224 but was only increased by 0.87-fold relative to YK193. Finally, the xylose consumption rate of YK197 at 24 h was 0.68-fold higher than that of SS118. These changes in the xylose consumption rate at 24 h in the presence of PPP gene overexpression are summarized in a schematic drawing (Fig. 4). YK246, which co-expressed three PPP genes (Km*RKI1*- Km*TAL1*-Sc*TKL1*), displayed the highest xylose consumption rate at 24 h (1.03 g/L/h) among the yeast strains tested in this study, and produced an ethanol yield of 0.45 g-ethanol/g-total sugars.

### Comparison of XR-XDH with XI, the effect of *PHO13* deletion and overexpression of all PPP genes on glucose/xylose co-fermentation

To reveal the effect of sugar composition of the fermentation media, we examined and compared the effect of *PHO13* disruption and overexpression of all PPP genes on glucose/xylose co-fermentation (85 g/L d-glucose, 35 g/L d-xylose, Additional file 3: Fig. S1) and xylose fermentation (60 g/L d-xylose, Additional file 4: Fig. S2) at 36 °C using the recombinant *S. cerevisiae* strains YK001 (XR-XDH), YK002 (XR-XDH, *pho13*Δ), YK115 (XR-XDH, *pho13*Δ::Sc*RKI1*-Sc*RPE1*-Sc*TAL1*-Sc*TKL1*), SS82 (XI), YK149 (XI, *pho13*Δ) and YK150 (XI, *pho13*Δ:Sc*RKI1*-Sc*RPE1*-Sc*TAL1*-Sc*TKL1*). A control strain with XR-XDH driven by the TDH3 promoter (YK001) assimilated d-glucose completely within 24 h, but the concentration of residual d-xylose was about 12 g/L at 72 h. The concentration of ethanol produced from d-glucose to d-xylose reached about 45 g/L at 72 h. On the other hand, the kinetics of d-glucose and d-xylose consumption of the control strain with XI (SS82) were similar to those for YK001 (XR-XDH), but the concentration of ethanol produced at 72 h was much higher at about 48.5 g/L. The production of xylitol and glycerol by SS82 was less than that by YK001 (xylitol: 2.8-fold, glycerol: 1.5-fold). Deletion of *PHO13* decreased the ethanol yields in both XR-XDH- (YK001) and XI-containing (YK149) strains at 72 h (YK002: 0.28 g/g, YK149: 0.33 g/g) on glucose/xylose co-fermentation. Furthermore, overexpression of four PPP genes in addition to the deletion of *PHO13* resulted in decreased ethanol yields in both XR-XDH and XI strains at 72 h (YK115: 0.13 g/g, YK150: 0.29 g/g) compared to strains that had only the *PHO13* deletion (YK002 and YK149) and control strains (YK001 and SS82). The strain expressing XI produced higher ethanol yields than did XR-XDH. Thus, *PHO13* disruption and overexpression of all PPP genes in these strains with the presence of XR-XDH and XI for xylose metabolism did not improve ethanol yield or xylose consumption rate in YPDX medium at 36 °C.

## Discussion

For the commercial production of bioethanol from cellulosic materials, development of microorganisms capable of highly efficient xylose fermentation is critical. To achieve this objective, many studies have demonstrated that expression of PPP genes can improve xylose metabolism (Kuyper et al. 2005; Karhumaa et al. 2007; Lee et al. 2014; Qi et al. 2015). We recently showed that the best combination of PPP gene overexpression in the *S. cerevisiae* haploid strain IR-2 that carries XR-XDH genes depended on the fermentation temperature in 60 g/L xylose medium (Kobayashi et al. 2017). For the SSCF process, one of the most economical fermentation processes to produce ethanol from cellulosic material at an industrial scale, the preferable temperature is higher than that for ethanologenic microorganisms. In addition, simultaneous consumption of d-glucose and d-xylose is desirable for economical ethanol production from cellulosic biomass. The concentrations of sugars in the raw material and the utilization of these sugars during the course of fermentation are also important factors for economical ethanol production, because changes in the concentrations of these sugars during glucose/xylose co-fermentation can alter gene expression levels in the microorganisms. For these reasons, it is critical to optimize expression levels of non-oxidative PPP genes under conditions that mimic those in the industrial process rather than experimental conditions such as low temperature growth on d-xylose-containing medium that was used for our previous study. However, the effects of overexpression of PPP genes on glucose/xylose co-fermentation, particularly at higher temperature, of *S. cerevisiae* IR-2 expressing XI were unknown.

In the present study, we systematically investigated how overexpression of PPP genes derived from *S. cerevisiae* and thermotolerant yeast *Kluyveromyces marxianus* expressed either singly or in combination by a *S. cerevisiae* strain harboring the XI gene affected glucose/xylose co-fermentation at 36 °C. The concentration of sugars, 85 g/L d-glucose and 35 g/L d-xylose, used in the fermentation experiments are based on typical sugarcane bagasse saccharified solutions. Among strains overexpressing a single PPP gene, *TKL1* (YK185) or *RKI1* (YK183) showed a dramatically increased d-xylose consumption rate at 24 h on glucose/xylose co-fermentation, whereas *TAL1* (YK184) and *RPE1* (YK186) did not (Fig. 1 and Table 2, also summarized in Fig. 4). Further experiments with yeast expressing multiple PPP genes clearly showed an optimal combination of PPP genes that could be used to improve xylose fermentation at high temperature: overexpression of *TKL1* from *S. cerevisiae* with *RKI1* and *TAL1* from *K. marxianus* produced the highest rates of xylose consumption and ethanol productivity among the strains examined (YK246, Figs. 2, 3 and Table 2, also summarized in Fig. 4). This result may appear to be inconsistent with a previous report suggesting that the efficiency of xylose fermentation should be attributed, at least in part, to increased *TAL1* expression in a *S. cerevisiae* strain expressing XI obtained by an adaptive evolution experiment (Vilela Lde et al. 2015). This study suggested that the amount of intermediary metabolites would differ between glucose/xylose co-fermentation at 36 °C and xylose fermentation alone at 30 °C. Other studies also reported that overexpression of four PPP genes could improve xylose metabolism (Kuyper et al. 2005; Qi et al. 2015). However, comparisons of our results with these previous studies is difficult due to differences in the experimental conditions, including genes for xylose metabolism, genetic background of the parental strain, fermentation temperature, inoculum dose and sugar concentrations. Thus, several points raised by our results should be addressed by future studies, including: (i) the suggestion that sedoheptulose 7-phosphate levels will be deficient in strains that do not overexpress *RKI1* and *TKL1* during glucose/xylose co-fermentation at high temperature because TKL1 synthesizes sedoheptulose 7-phosphate and glyceraldehyde 3-phosphate from xylose 5-phosphate and ribose 5-phosphate; and (ii) whether the synthesis of ribose 5-phosphate from ribulose 5-phosphate by RKI1 is insufficient. Overexpression of *RPE1* may not be needed to increase the d-xylose consumption rate on glucose/xylose co-fermentation at a high temperature as its gene product could induce competition of enzyme reactions between xylulose 5-phosphate from xylose to ribulose 5-phosphate from glucose. In fact, only slight differences in d-xylose consumption rates at 24 h and ethanol production at 72 h were seen between YK246 and YK193 (Fig. 1 and Table 2, also summarized in Fig. 4). Thus, the present study clearly showed that overexpression of the PPP genes Km*RKI1*, Km*TAL1* and Sc*TKL1* provided the best performance for xylose consumption and ethanol production on glucose/xylose co-fermentation at high temperature, indicating that optimizing xylose metabolism according to the individual and realistic experimental conditions, especially fermentation temperature, type and concentration of sugars and cell growth during fermentation, will be important for generating strains that have the best performance under these conditions.

To improve xylose metabolism, factors other than PPP may also need to be considered. Elimination of NAD-dependent glycerol 3-phosphate dehydrogenase (*GPD*) that has two homologous genes, *GPD1* and *GPD2*, which are involved in glycerol metabolism by *S. cerevisiae* (Eriksson et al. 1995), increased ethanol yield while decreasing glycerol production (Björkqvist et al. 1997; Henningsen et al. 2015). In a preliminary examination, we verified that SS118 (XI *gpd2*Δ) exhibited decreased glycerol production and improved ethanol production (Seike et al. unpublished observations). As such, in the present study, each PPP gene was inserted in the *GPD2* locus. As expected, glycerol production in all the strains was reduced compared with the parental strain SS82 (Additional file 2: Table S2).

On the other hand, whether deletion of *PHO13*, a putative alkaline phosphate gene, improves xylose fermentation is controversial. Several studies showed that deletion of *PHO13* improves xylose metabolism in *S. cerevisiae* expressing XR-XDH. The improvement yielded by *PHO13* deletion could be due to altered redox levels on d-xylose (Van Vleet et al. 2008) and xylulose-5-phosphate phosphatase (Kim et al. 2013) that induce a global transcriptional response (Kim et al. 2015). The deletion of *PHO13* had a beneficial effect on fermentation with improved activity of the XI pathway, and high ethanol yield (0.45 g/g) in 40 g/L d-xylose medium at 30 °C (Lee et al. 2014). Another study demonstrated that disruption of *PHO13* enhanced the d-xylose consumption rate (0.31 g/g-cell/h), ethanol productivity (0.11 g/g-cell/h) and ethanol yield (0.45 g/g) in an XI-expressing, xylose-fermenting yeast strain under oxygen-limited conditions in 50 g/L d-xylose at 30 °C (Bamba et al. 2016). In contrast to these reports, deletion of *PHO13* in *S. cerevisiae* expressing XI sharply weakened growth on xylose plates (Shen et al. 2012) and promoted accumulation of 4-phospho-erythronate and 6-phospho-gluconate (Collard et al. 2016). Based on these earlier results, we examined and evaluated the effect of deletion of *PHO13* and overexpression of all PPP genes on a glucose/xylose mixed medium or only xylose medium at 36 °C in the recombinant *S. cerevisiae* strains YK001 (XR-XDH), YK002 (XR-XDH *pho13*Δ), YK115 (XR-XDH *pho13*Δ:Sc*RKI1*-Sc*RPE1*-Sc*TAL1*-Sc*TKL1*), YK149 (XI *pho13*Δ) and YK150 (XI *pho13*Δ:Sc*RKI1*-Sc*RPE1*-Sc*TAL1*-Sc*TKL1*). *S. cerevisiae* strains expressing XR-XDH consumed 85 g/L d-glucose within 24 h but had incomplete consumption of 35 g/L d-xylose even at 72 h on glucose/xylose co-fermentation at 36 °C when cells were initially inoculated at OD_600_ = 3 (Additional file 3: Figure S1). Notably, no difference was observed between the parental (YK001, SS82) and *PHO13*Δ (YK002, YK149) strains, but xylose consumption was decreased in the strain that overexpressed all four PPP genes (YK115, YK150) on glucose/xylose co-fermentation (Additional file 3: Figure S1). Incidentally, the concentrations of ethanol production of YK001 and SS82 were about 45.1 g/L and 48.6 g/L at 72 h, respectively. Ethanol production by strains carrying XI could have been higher than those with XR-XDH due to a decrease in the by-product xylitol compared with xylose consumption. Although *S. cerevisiae* strains expressing XI can directly convert d-xylose to d-xylulose without a redox reaction, *GRE3* encoding an intrinsic aldo-keto reductase and producing xylitol should be deleted to reduce xylitol formation (Yamanaka. 1969; Träff et al. 2001).

In xylose fermentation, however, markedly different results were obtained for strains with XR-XDH and XI. For strains harboring XR-XDH, xylose metabolism was clearly improved by deletion of *PHO13* (YK002) and additional overexpression of the four PPP genes (YK115) as expected. For strains harboring XI, however, xylose consumption was decreased by the deletion of *PHO13* and recovered to control levels by overexpression of the four PPP genes (Additional file 4: Figure S2, Kobayashi et al. 2017). Ismail et al. (2013) showed that the expression of several genes was up- or downregulated by more than twofold at 38 °C compared to at 30 °C in *S. cerevisiae* carrying XR-XDH. These results suggested that PPP could be a limiting step during ethanol production from d-xylose at high temperature when severely downregulated PPP gene expression might result in accumulation of certain intermediates that in turn affect the metabolic flux in PPP. Whatever the case, our results indicate that *PHO13* deletion should not be adopted due to its negative effect on strains carrying XI.

**Fig. 5:**
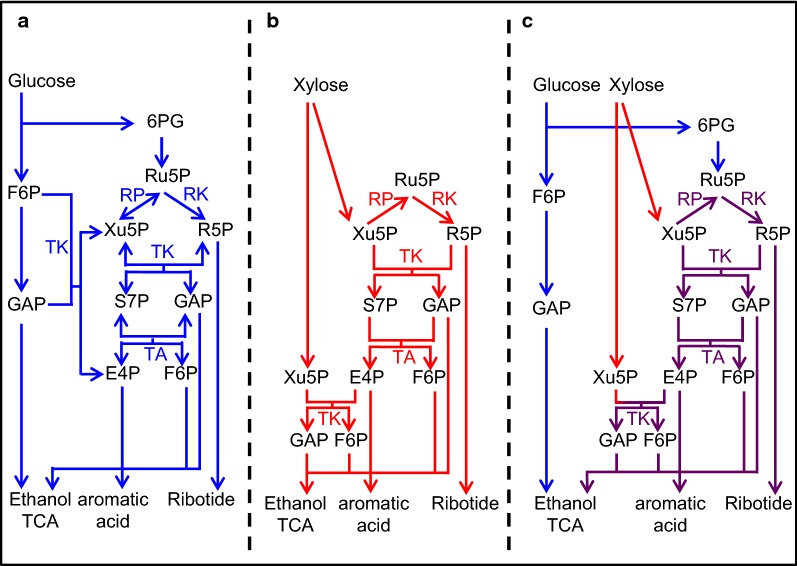
Differences in metabolic pathways for production of ethanol and energy supply with glucose, xylose and mixed sugars fermentation. Ethanol, aromatic acids and ribotides are synthesized from glucose (blue lines), xylose (red lines), or both glucose and xylose (purple lines) on glucose fermentation (**a**), xylose fermentation (**b**), and glucose/xylose co-fermentation (**c**) via the glycolytic pathway and pentose phosphate pathway

Developing an ideal strain for cellulosic ethanol production is challenging due to the complex nature of glucose/xylose co-fermentation. The entire fermentation process can be divided into three phases: (i) glucose assimilation (on catabolite repression), (ii) simultaneous assimilation of glucose and xylose (off catabolite repression), and (iii) subsequent assimilation of residual xylose. The interphase transitions can dramatically affect the expression of various metabolic genes such as hexose transporters since activities of gene promoters can be altered by extracellular concentration and uptake of sugars (Sedlak and Ho 2004). Here, we used the well-characterized strong promoter TDH3 and CYC1 terminator to control expression of PPP genes. Future studies should examine suitable promoters for the regulation of the expression of individual PPP genes to provide the necessary levels of PPP enzymes that promote harmonized xylose metabolic flux. Taken together, the activities of PPP enzymes required for ideal xylose metabolism differ according to glucose/xylose co-fermentation and for xylose fermentation at high temperature (Fig. 5).

## Additional files

**Additional file 1: Table S1.** Oligonucleotide sequences used for synthesis of DNA fragments for homologous recombination.

**Additional file 2: Table S2.** Metabolic characteristics of yeast strains grown in YPDX medium.

**Additional file 3: Figure S1.** Fermentation profiles of XR-XDH-inserted and XI-inserted strains with *pho13*Δ on glucose/xylose co-fermentation.

**Additional file 4: Figure S2.** Fermentation profiles of XR-XDH-inserted and XI-inserted strains with *pho13*Δ on xylose containing medium.

## Abbreviations

PPP: pentose phosphate pathway
*S. cerevisiae or* Sc: *Saccharomyces cerevisiae*
*TAL1*: transaldolase
*TKL1*: transketolase
*RKI1*: ribose-5-phosphate ketol-isomerase
*RPE1*: d-ribulose-5-phosphate 3-epimerase
XI: xylose isomerase
XR or *XYL1*: NADPH-dependent xylose reductase
XDH or *XYL2*: NAD^+^-dependent xylitol dehydrogenase
*PHO13*: putative alkaline phosphate
*HXT7*: xylose-permeable hexose transporter
SSCF: simultaneous saccharification and co-fermentation
*K. marxianus* or Km: *Kluyveromyces marxianus*
*GRE3*: aldo–keto reductase
Lp: *Lachnoclostridium phytofermentans*
*AUR1*: phosphatidylinositol:ceramide phosphoinositol transferase (Aureobasidin A resistance gene)
LiOAc: lithium acetate
DNA: deoxyribonucleic acid
HPLC: high performance liquid chromatography
RI: refractive index
*Opt*: codon-optimized
*GPD1* and *GPD2*: NAD-dependent glycerol 3-phosphate dehydrogenase
CYC1: Cytochrome c, isoform 1
TDH3: Glyceraldehyde-3-phosphate dehydrogenase (GAPDH), isozyme 3
