# Supplementary material for: Systematic optimization of gene expression of pentose phosphate pathway enhances ethanol production from a glucose/xylose mixed medium in a recombinant Saccharomyces cerevisiae
Source: AMB Express. 2018 Aug 27;8:139. doi: 10.1186/s13568-018-0670-8 (PMC6111014; doi:10.1186/s13568-018-0670-8)
Supplement: Supplementary file 1 — Additional file 1: Table S1. Oligonucleotide sequences used for synthesis of DNA fragments for homologous recombination. [file 13568_2018_670_MOESM1_ESM.docx]

Additional file 1: Table S1. Oligonucleotide sequences used for synthesis of DNA fragments for homologous recombination.

| Gene | Direction | 5'→3' |
| --- | --- | --- |
| GPD2Δ | for1 | tttccttcgctccccttccttatcaACACAGGAAACAGCTATGACCATGA |
|  | for2 | agattcaattctctttccctttccttttccttcgctccccttccttatca |
|  | rev1 | aagaggcaacaggaaagatcagagggCGACAGCAGTATAGCGACCAGCAT |
|  | rev2 | ataatgataaattggttgggggaaaaagaggcaacaggaaagatcagagg |
| PHO13Δ | for1 | gcttgccctgacaaagaatatacaactcgggaaaACACAGGAAACAGCTA |
|  | for2 | aaaaaaaagccttatagcttgccctgacaaagaatatacaactcgggaaa |
|  | rev1 | agtaattctaccccaagattttgcattgctcctAATCGACAGCAGTATAG |
|  | rev2 | atttttccttttcaaaaagtaattctaccccaagattttgcattgctcct |

Uppercase letters indicate sequences corresponding to the gene to be amplified; lowercase letters indicate sequence overhangs for homologous recombination
