# Supplementary material for: Systematic optimization of gene expression of pentose phosphate pathway enhances ethanol production from a glucose/xylose mixed medium in a recombinant Saccharomyces cerevisiae
Source: AMB Express. 2018 Aug 27;8:139. doi: 10.1186/s13568-018-0670-8 (PMC6111014; doi:10.1186/s13568-018-0670-8)
Supplement: Supplementary file 2 — Additional file 2: Table S2. Metabolic characteristics of yeast strains grown in YPDX medium. [file 13568_2018_670_MOESM2_ESM.docx]

Additional file 2: Table S2. Metabolic characteristics of yeast strains grown in YPDX medium.

|  | h | Glucose  (g/L) | Xylose  (g/L) | Xylitol  (g/L) | Glycerol  (g/L) | Acetate  (g/L) | Ethanol  (g/L) |
| --- | --- | --- | --- | --- | --- | --- | --- |
| SS82 | 24 | N/D | 18.75±3.20 | N/D | 5.00±0.34 | 1.28±0.31 | 43.94±1.93 |
|  | 48 | N/D | 12.44±2.90 | 0.52±0.90 | 5.27±0.28 | 1.41±0.31 | 47.06±1.84 |
|  | 72 | N/D | 8.84±2.65 | 1.51±0.14 | 5.32±0.27 | 1.50±0.30 | 48.60±1.73 |
| SS118 | 24 | N/D | 20.97±1.62 | 0.47±0.82 | 4.04±0.12 | 1.07±0.19 | 43.18±1.43 |
|  | 48 | 0.30±0.52 | 13.58±3.10 | 0.59±1.02 | 4.21±0.13 | 1.17±0.19 | 47.20±1.43 |
|  | 72 | N/D | 10.19±3.64 | 1.09±1.00 | 4.31±0.07 | 1.25±0.19 | 48.07±1.54 |
| YK184 | 24 | N/D | 21.71±0.79 | 0.34±0.29 | 3.91±0.04 | 0.83±0.10 | 44.24±0.27 |
|  | 48 | N/D | 14.10±2.11 | 0.90±0.19 | 3.95±0.15 | 0.93±0.09 | 47.19±0.12 |
|  | 72 | N/D | 10.57±3.17 | 1.30±0.04 | 4.11±0.07 | 1.02±0.06 | 49.30±1.02 |
| YK185 | 24 | N/D | 13.18±0.90 | N/D | 4.32±0.16 | 1.26±0.03 | 48.19±2.13 |
|  | 48 | N/D | 4.79±1.12 | 0.89±0.77 | 4.41±0.11 | 1.36±0.02 | 51.23±2.22 |
|  | 72 | N/D | 2.26±0.53 | 1.01±0.87 | 4.50±0.06 | 1.50±0.02 | 52.62±1.82 |
| YK183 | 24 | N/D | 17.69±1.78 | 0.44±0.77 | 4.05±0.22 | 1.02±0.27 | 46.65±1.76 |
|  | 48 | N/D | 8.17±2.88 | 0.79±0.78 | 4.19±0.19 | 1.10±0.24 | 50.64±2.09 |
|  | 72 | N/D | 4.23±2.64 | 0.89±0.82 | 4.31±0.10 | 1.17±0.23 | 53.14±1.00 |
| YK186 | 24 | N/D | 19.77±0.61 | 0.50±0.44 | 3.97±0.07 | 0.99±0.01 | 43.65±1.01 |
|  | 48 | N/D | 12.59±1.59 | 0.70±0.61 | 4.14±0.08 | 1.10±0.02 | 46.79±0.80 |
|  | 72 | N/D | 8.52±2.16 | 0.90±0.78 | 4.18±0.09 | 1.18±0.01 | 48.67±1.07 |
| YK223 | 24 | N/D | 17.37±1.27 | N/D | 4.03±0.03 | 0.83±0.06 | 45.19±0.89 |
|  | 48 | N/D | 7.13±1.79 | 0.37±0.64 | 4.20±0.03 | 0.92±0.07 | 49.92±1.39 |
|  | 72 | N/D | 3.12±1.29 | 0.42±0.72 | 4.23±0.07 | 0.99±0.08 | 51.33±0.99 |
| YK193 | 24 | N/D | 12.01±1.19 | N/D | 4.41±0.20 | 1.34±0.03 | 47.84±0.72 |
|  | 48 | N/D | 3.10±0.86 | N/D | 4.56±0.12 | 1.45±0.01 | 51.86±0.47 |
|  | 72 | N/D | 1.55±0.33 | N/D | 4.66±0.17 | 1.56±0.02 | 53.46±0.74 |
| YK224 | 24 | N/D | 16.71±1.76 | 0.56±0.48 | 3.96±0.12 | 0.89±0.06 | 45.37±0.38 |
|  | 48 | N/D | 7.30±1.52 | 0.74±0.64 | 4.08±0.17 | 1.00±0.06 | 49.98±0.54 |
|  | 72 | N/D | 3.23±1.19 | 0.81±0.71 | 4.17±0.11 | 1.09±0.08 | 51.47±0.13 |
| YK246 | 24 | N/D | 10.42±1.42 | N/D | 4.90±0.05 | 0.90±0.02 | 49.76±0.67 |
|  | 48 | N/D | 2.38±0.81 | N/D | 5.03±0.08 | 0.97±0.02 | 54.00±1.05 |
|  | 72 | N/D | 0.96±0.35 | N/D | 5.15±0.08 | 1.06±0.03 | 54.33±0.55 |
| YK247 | 24 | N/D | 16.80±2.17 | N/D | 4.82±0.16 | 0.97±0.04 | 47.34±2.42 |
|  | 48 | N/D | 6.99±2.36 | N/D | 4.87±0.08 | 1.05±0.06 | 51.26±1.33 |
|  | 72 | N/D | 3.54±1.48 | N/D | 4.96±0.10 | 1.14±0.05 | 52.67±1.01 |
| YK248 | 24 | N/D | 15.11±0.63 | N/D | 4.90±0.08 | 1.01±0.03 | 48.54±0.96 |
|  | 48 | N/D | 5.67±0.50 | N/D | 4.91±0.07 | 1.06±0.03 | 51.80±1.14 |
|  | 72 | N/D | 3.09±0.49 | N/D | 4.97±0.08 | 1.14±0.01 | 53.00±0.84 |
| YK249 | 24 | N/D | 15.36±0.85 | N/D | 4.61±0.02 | 0.93±0.02 | 48.28±0.33 |
|  | 48 | N/D | 5.82±1.18 | N/D | 4.86±0.04 | 1.00±0.01 | 53.03±1.10 |
|  | 72 | N/D | 2.77±1.03 | N/D | 4.90±0.06 | 1.08±0.02 | 54.27±1.86 |
| YK197 | 24 | N/D | 25.17±1.36 | N/D | 4.55±0.10 | 1.35±0.00 | 43.29±0.52 |
|  | 48 | N/D | 19.16±2.52 | 1.70±0.03 | 4.63±0.07 | 1.43±0.00 | 46.04±1.18 |
|  | 72 | N/D | 16.10±2.18 | 1.95±0.04 | 4.79±0.09 | 1.54±0.01 | 48.41±1.29 |
| YK001 | 24 | 0.08±0.13 | 18.22±2.22 | 3.22±0.23 | 7.74±0.07 | 1.94±0.40 | 42.52±1.10 |
|  | 48 | N/D | 13.42±3.72 | 4.06±0.20 | 8.03±0.08 | 2.02±0.39 | 44.24±1.28 |
|  | 72 | N/D | 12.07±3.88 | 4.25±0.21 | 8.11±0.23 | 2.10±0.43 | 45.11±1.50 |
| YK002 | 24 | 0.09±0.16 | 19.54±0.86 | 3.09±0.22 | 8.17±0.04 | 2.32±0.30 | 42.13±1.27 |
|  | 48 | N/D | 15.16±3.55 | 3.90±0.26 | 8.22±0.10 | 2.43±0.34 | 42.75±1.74 |
|  | 72 | N/D | 14.44±4.21 | 4.15±0.45 | 8.47±0.09 | 2.55±0.33 | 43.63±1.56 |
| YK115 | 24 | 0.20±0.17 | 27.15±3.04 | 1.63±0.84 | 7.42±0.80 | 2.09±0.49 | 39.83±0.04 |
|  | 48 | 0.03±0.03 | 26.51±3.15 | 1.82±0.89 | 7.42±0.83 | 2.21±0.48 | 39.79±0.19 |
|  | 72 | N/D | 25.72±2.90 | 1.86±0.87 | 7.26±0.99 | 2.25±0.50 | 40.09±0.53 |
| YK149 | 24 | 0.80±0.00 | 19.25±0.74 | 1.46±0.15 | 5.71±0.14 | 1.84±0.03 | 43.88±0.74 |
|  | 48 | N/D | 13.36±0.22 | 1.90±0.02 | 5.84±0.05 | 1.99±0.01 | 45.86±0.22 |
|  | 72 | N/D | 10.01±0.30 | 2.10±0.03 | 5.95±0.04 | 2.11±0.02 | 47.17±0.13 |
| YK150 | 24 | N/D | 22.36±0.20 | 1.15±0.24 | 5.45±0.19 | 1.67±0.04 | 43.09±1.07 |
|  | 48 | N/D | 17.29±1.42 | 1.74±0.01 | 5.50±0.13 | 1.82±0.02 | 44.50±0.74 |
|  | 72 | N/D | 13.85±1.87 | 2.00±0.04 | 5.59±0.11 | 1.98±0.01 | 46.01±1.07 |

N/D: not detected
