# Supplementary material for: Systematic optimization of gene expression of pentose phosphate pathway enhances ethanol production from a glucose/xylose mixed medium in a recombinant Saccharomyces cerevisiae
Source: AMB Express. 2018 Aug 27;8:139. doi: 10.1186/s13568-018-0670-8 (PMC6111014; doi:10.1186/s13568-018-0670-8)
Supplement: Supplementary file 3 — Additional file 3: Figure S1. Fermentation profiles of XR-XDH-inserted and XI-inserted strains with pho13Δ on glucose/xylose co-fermentation. [file 13568_2018_670_MOESM3_ESM.pdf]

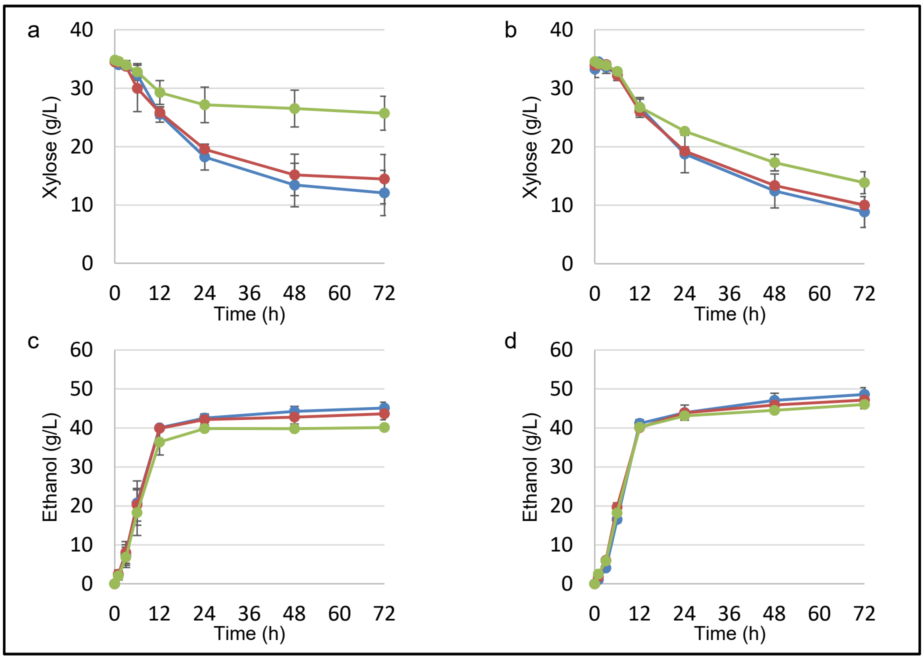


**Figure S1. Fermentation profiles of XR-XDH-inserted and XI-inserted strains with *pho13δελτα* on glucose/xylose co-fermentation.**

Cells were initially inoculated at $OD_{600}$=3 and cultivated in 85 g/L glucose and 35 g/L xylose for 72 hours at 36 ° C. **a, b**: concentration of D-xylose; **c, d**: concentration of ethanol; **a, c**: *S. cerevisiae* expressing XR-XDH; **b, d**: *S. cerevisiae* expressing XI. Blue line: control strains (YK001 or SS82). Red line: strains with *PHO13* deletion (YK002 or YK149); Green line: strains overexpressing all PPP genes with *PHO13* deletion (YK115 or YK150). Each data point is an average of triplicate cultures.
