# Supplementary material for: Systematic optimization of gene expression of pentose phosphate pathway enhances ethanol production from a glucose/xylose mixed medium in a recombinant Saccharomyces cerevisiae
Source: AMB Express. 2018 Aug 27;8:139. doi: 10.1186/s13568-018-0670-8 (PMC6111014; doi:10.1186/s13568-018-0670-8)
Supplement: Supplementary file 4 — Additional file 4: Figure S2. Fermentation profiles of XR-XDH-inserted and XI-inserted strains with pho13Δ on xylose containing medium. [file 13568_2018_670_MOESM4_ESM.pdf]

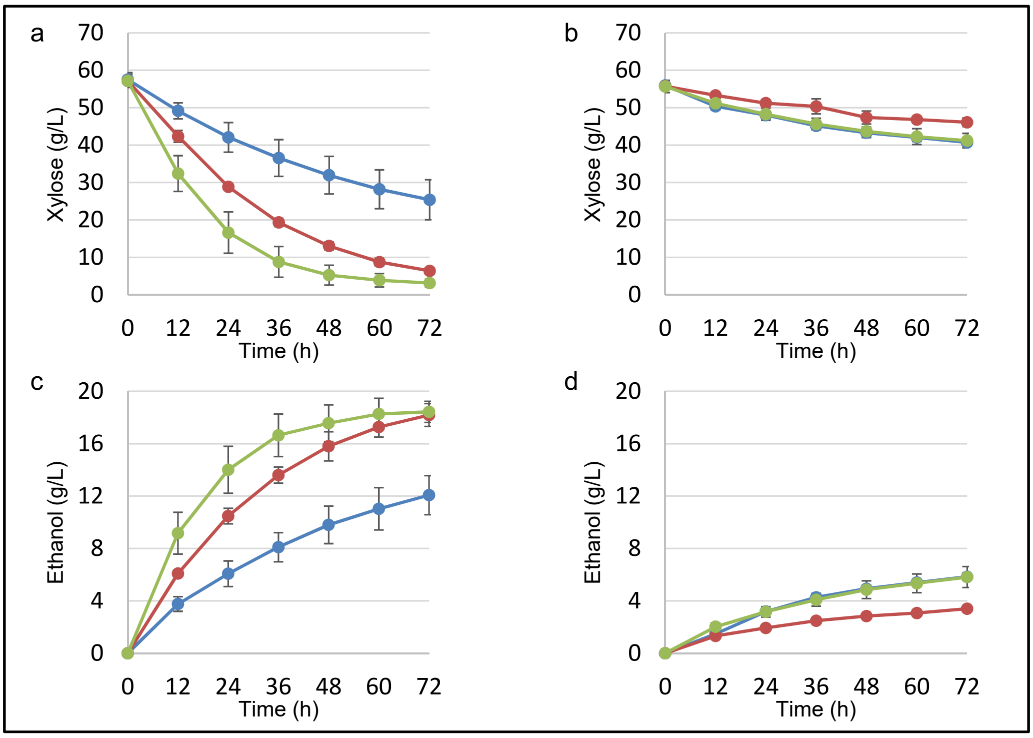

Figure S2. Fermentation profiles of XR-XDH-inserted and XI-inserted strains with *pho13delta* on xylose containing medium. Cells were initially inoculated at OD_{600}=5 and cultivated in 60 g/L xylose for 72 hours at 36 ° C. **a, b**: concentration of D-xylose; **c, d**: concentration of ethanol; **a, c**: *S. cerevisiae* expressing XR-XDH; **b, d**: *S. cerevisiae* expressing XI. Blue line: control strains (YK001 or SS82); Red line: strains with *PHO13* deletion (YK002 or YK149); Green line: strains overexpressing all PPP genes with *PHO13* deletion (YK115 or YK150). Each data point is an average of triplicate cultures.
